# On the Pathology and Treatment of Scrofula

**Published:** 1847-01-01

**Authors:** 


On the Pathology and Treatment of Scrofula; being the Fothergillian Prize Essay for 1846. By *Robert Mortimer Glover*, M.D., &c., Lecturer on Materia Medica in the Newcastle Medical School. 8vo. pp. 315, with 4 Plates. Churchill, London, 1846.

Within the last two years, we have devoted so much space to the consideration of the very important subject of Scrofula, in reviewing the valuable works of Lugol, Dr. Tyler Smith, Messrs. Rilliet and Barthez, and lastly of Mr. Phillips,* that neither our readers, nor Dr. Glover himself, will expect that we should give an extended analysis of his work. Before proceeding to notice those parts of it which have chiefly attracted our attention, it is but fair to the author to state, that it was in the process of publication when Mr. Phillips' recent treatise was published.

Dr. G. divides his work into two parts, one devoted to the consideration of the Pathology, the other to that of the Treatment, of Scrofula. The former contains eight, the latter two, Chapters.

Chapter I. is devoted to the "Description of Scrofulous and Tuberculous Matter." From this very expression, it will be at once perceived that our author regards these two forms of morbid deposit as essentially of the same nature; in opposition, therefore, to the opinion of Mr. Phillips on the subject, and in accordance with that which we expressed in our review of his work.

The tubercles of the lungs are not to be distinguished from those found in other tissues and organs of the body :—

"We have," says Dr. Glover, after quoting the sentiments of Barthez and Rilliet, Valleix, and other writers to the same effect, "observed, in granular meningitis, the forms of grey granulation and yellow particles answering very well to the yellow points which appear in the grey granulation of the lungs. The miliary tubercle may exist in all the organs, and it is in the form of a grey infiltrated matter, granular to the microscope, more or less diffused through the substance of a gland that we detect the first appearance of mesenteric tubercle; afterwards we find a more crude or yellow appearance of tubercle matter, as in the lungs. The appearance of infiltrated grey matter is especially marked in effusions organized between the tunics of the intestinal canal. Some of the illustrations which are appended shew the tubercular effusion in a mesenteric gland; 1stly, in a diffused form throughout the hypertrophied tissue of the organ; 2ndly, forming striæ and patches varying in hue from grey to yellow; 3rdly, in cysts filled either

---

* Vide the Numbers of the Medico-Chirurgical Review for January and October 1845, and July 1846.

with a tuberculous powder or with a curdy matter; 4thly, in masses of lardaceous consistence implicating either the whole gland, or more or less of its structure. We have specimens of bronchial glands and bronchi sprinkled over, as it were, with a tuberculous powder, and studded with cretaceous fragments.

"High authorities, notwithstanding, have spoken of the grey granulation as a form of tubercle peculiar to the lungs, but we repeat as Barthez and Rilliet observe; 'it exists in all the organs, not only in the intestines, peritoneum, and pleura, but in the spleen, the liver, the kidneys, the lymphatic glands and the meninges.' "* P. 29.

He subsequently remarks:—

"It is exceedingly probable that the grey and gelatiniform infiltrations of Laennec are merely early stages of the tubercular effusion. According to this author, the gelatiniform effusion similar to an œdema, formed of a viscous lymph, passes into the grey infiltration, as it is probable that this latter, like the grey granulation, passes into the yellow tubercle." P. 30.

In spite of the dissenting opinions of certain writers, we cannot but regard tuberculous deposit as essentially non-vascular in its structure, and altogether as an unorganized product or formation; being destitute of true nucleated cells, and consisting chiefly of molecular, irregularly-shaped granules and corpuscles.

Is there any occasion for dwelling upon the Microscopic Characters of Tubercle? We fear not; for, notwithstanding the many elaborate descriptions that have been published, it must be confessed, we fear, that but little satisfactory information has been obtained. In our review of Mr. Phillips' work, will be found an account of the examinations made by Messrs. Dalrymple and Gulliver, two good authorities upon the subject; and, in a preceeding number of this Journal, the reader will find M. Lebert's description, which is generally received as one of the most accurate that has yet been published.

Dr. Glover has given extracts from the writings of Canstatt, Vogel, Scherer, and some other continental microscopists. For these we must refer the curious reader to his work. His concluding observations are as follow:—

"Under the microscope, undoubtedly, tubercular masses exhibit evidences of a deficiency of formative power, when compared with the organized deposits of ordinary inflammation on the one hand, and those of a parasitic character on the

---

* According to our author's experience, tuberculous deposits are generally to be found between the investing membranes and the parenchymatous substance of the viscera. "In a specimen of tubercle of the heart, which we recently examined, all the tubercles were in contact with the endocardium or pericardium, although some of them had pushed completely through the muscular substance. In the tubercles of the spleen represented in the illustrations, the morbid formations are connected with the investing membrane of the organ. In all the specimens of tubercle of the kidney which we have seen, the deposit was connected either with the mucous membrane lining the ducts of the organ, or with the external covering. In the same way with the brain, we generally find the tubercles in contact with the external or internal membranes. And so with the bones, the tubercles are in relation with either the periosteum or the medullary ramifications. Not that we should deny the existence of these morbid formations in the parenchyma itself of an organ." P. 231.

other. In these latter, the formative power is not sufficiently controlled by the laws of the general system, nor directed, as in ordinary inflammation, to a reparative function, but the new formed product takes on itself an independent vitality. In scrofulous and tubercular formations the formative power is deficient, and the cells which are formed, either remain abortive, or instead of cells, we find the granular corpuscles already described. The cells which many have found, belong most frequently, in our opinion, to original structures mixed up with the tubercle-masses: thus in fig. 4 there is a microscopic representation of lung tubercle, shewing the remains of vascular and cellular tissue, mixed with the usual tubercular granules; the cells in fig. 7, from tubercle of the heart, are probably of the same character, and similar to those figured by Scherer, as already stated. We have not been able to discover any essential difference between the microscopic structure of infiltrated tubercle and that of the ordinary tubercle already described. The structure of infiltrated tubercle consists of irregular corpuscles and granules, some epithelial scales mixed with a few exudation corpuscles, and a great number of minute molecules." P. 53.

If the Microscopic History of Tubercle has been hitherto so barren of available information, much more so, in this respect, is the Chemical, notwithstanding the labours of many eminent analysts. Those, who are interested in tracing the results obtained by successive experimenters, may be gratified with the details in our author's work. We shall content ourselves with giving one extract.

"The results of the chemical analysis of tubercle, and its after-products, of scrofulous bones, &c., although they may not as yet warrant very decisive conclusions, yet furnish some useful information, which will be found to bear upon the pathological propositions advanced concerning the essential nature of scrofulous and tubercular affections.

"Thus the large quantity of fat and extractive matters in tubercle, has a direct bearing upon the theory supported by many of the advocates of the use of cod-liver oil in the treatment of these diseases. The existence of pyin is important, and could we be sure of that of casein in quantity, we might to a certain extent explain the unorganizability of tubercle. But we have never been able to satisfy ourselves that the protein constituent of tubercle, as examined by us, approaches much nearer to casein, than to albumen. Nevertheless, the researches of Preuss, Boudet, Scherer, and others, must be held decisive of the existence, at least in some cases, of casein; although the last-named observer is far from confirming former writers in the statement of a large proportion of tubercle matter being composed of this substance. We have made other examinations for casein than those recorded, and have never been able to detect its presence. Whoever considers the very doubtful power of the tests which we possess, for distinguishing these different substances in the animal body, will be very doubtful of the precise nature of the protein basis of tubercle. Nevertheless, we may perhaps conclude that there is great probability of this protein compound having a certain approach to casein, or at least of a portion of it, exhibiting a tendency to take on the characters of this latter substance." P. 88.

Chap. II. is entitled, "Humoral Pathology of Scrofula," and is occupied with the examination of the state of the Blood, Bile, Lymph, Chyle, and the Gastric and Urinary Secretions, in this disease. Dr. Glover seems to have bestowed considerable attention upon the hæmatological phenomena of Scrofula; but the results of his experiments have not added much—and indeed we could not expect it—to our information. All that we gather from them is, that there is "an increase in the solids of the serum, and a diminution of the blood-globules, which is very nearly the alteration that has been long suspected to exist."*

There is sound truth in the following passage.

"Changes in the blood, more subtile than any which can be detected by our analyses, may play a most important part in the phenomena of disease; and as Bredow well remarks, 'if a peculiar condition of the scrofulous blood could not be proved by chemical or microscopical researches, still it would not necessarily follow that such a state does not exist, but only that the necessary re-agents were not known, and the microscopes employed not sufficiently powerful.' "† P. 97.

Perhaps this will be the most suitable place to notice the opinion of our author as to the *Essential Nature*, or proximate cause, *of Scrofula*. The definition of the disease, which he proposes, is to this effect:—we should premise that the pathognomonic character of Inflammation is declared to be "the occurrence of effusion of blood-plasma with preceding congestion;" whether these phenomena be accompanied with pain, heat, redness and swelling, or not.

"Scrofula is (speaking of the actual diseased process, not of the diathesis, which has been elsewhere described), a peculiar modification of inflammation, whereby the usual, or, as they may be termed, the normal products of this process are not evolved, but instead of them other materials, incapable of passing into the regular cell forms, and which constitute the substance already described under the name of scrofulous or tuberculous matter. The peculiarity of this formation, and the continuance of the scrofulous diathesis are the causes of the characters assumed by the various after-processes which result from the existence of tubercle." P. 181.

He expands the same idea in the following passage.

"It is probable, that if the effusions in scrofulous and in ordinary inflammation were examined in their primitive state, no difference in anatomical character would be detected. The opinion maintained by Mr. Addison, regarding the actual passage of corpuscles from the blood in the process of inflammatory exudation, seems not in accordance with analogy. The probability is in favour of such exudations being composed, in the beginning, of a plastic fluid, in which the different corpuscles characteristic of ordinary inflammatory organization are afterwards developed. A scrofulous effusion, then, from some deficiency of organic power, of innate susceptibility, does not pass into the higher organized forms. We have, as a result, either a granular mass, destitute altogether of cell forms, or only abortive attempts at cell formation." P. 185.

Does all this throw any light upon the real history of Scrofula? Dr. Glover seems to think that it bears upon the question as to the influence of hereditary predisposition to the disease: "If scrofula be merely a

---

* Dr. Glover hazards a, not very probable, conjecture in the following passage:

"We are strongly inclined to suppose that the original states of the blood in Scrofula, and in Gout, may present much similarity; perhaps there may be more fibrin in the blood of gouty patients; and that from a deficiency in assimilative power in the former disease, the excess of albuminous matter is excreted from the blood in the form of tubercle, as already described; whereas in gout, the assimilation is more complete, and the excess of nutriment is manifested in the form of uric acid in the destructive digestion of the tissues: but this is, in great part, hypothetical." P. 232.

† "Ueber die Scrofelsucht, s. 30."

modification of the inflammatory process arising from a peculiar cachexia, and exhibiting every stage of deviation from the usual standard, we perceive at once how exaggerated are the notions of the excessive power of the hereditary influence, entertained by Lugol."

We regret to observe that Dr. Glover has, with the view of discovering more as to the nature of tuberculous formations, deemed it either necessary or right to repeat Cruveilheir's experiments of trying artificially to induce the disease in animals.

" Among the arguments usually advanced in favour of the production of tubercles by inflammation is the formation of them by artificial means. We have produced them in this way in the rabbit and dog, chiefly in order to examine them by the microscope. The mode of experimenting adopted was by incising the trachea, and injecting a quantity of mercury downward into the lungs. The appearance of the bodies which resulted, the animal being killed at a period of from one to two months after the operation, was not externally unlike that of tubercle; little round whitish masses, more or less agglomerated; and each nodule with a globule of mercury in its centre, around which the exuded matter was formed. Pus existed in some parts around the artificial tubercles; but on examining the broken up bodies by the microscope, although the structure of portions did not appear very different from that of the irregular granules and corpuscles of tubercle, yet the exudation corpuscles were tolerably numerous and those toward the edges of the exudation fully formed. Numerous nucleated cells also were found mixed with the mass; in short, the formation more nearly resembled an ordinary inflammatory product, such as we find in pulmonary hepatization." P. 196.

So much for the instruction afforded from this (as it seems to us) unwarrantable source.

It is not necessary to do more than only allude to the opinions, or rather mere conjectures, that have been put forth by certain writers as to the changes of the Gastric Juice, Bile, and Chyle in Scrofula. Some have even gone so far as to hold that the immediate cause of the disease is a deficient secretion of the bile! We were a good deal surprised to find that so intelligent a man as our author should express even his qualified assent to such a fanciful doctrine:

" Although we cannot recognize a merely deficient secretion of the fatty principles of the bile as the cause of scrofula, it is not very improbable that these states of the liver and bile are closely connected with the pathology of the disease." P. 120.

The only thing, that calls for notice in the chapter *On the Tuberculous Diathesis*, is the very just remark of Dr. Glover on the controverted point whether fair or black-haired persons are most subject to Scrofula:

" It might have been more satisfactory, if those who have made assertions on this head, as Dr. Alison in Edinburgh, Hufeland in Berlin, Lloyd in London, and Lugol in Paris, had informed us, first—what proportion persons of light complexion bear to those who are dark, in the inhabitants of these places, respectively; and second—whether the relation among people in general holds good in those affected with scrofula, or to what extent it is altered. This is clearly the only mode by which to arrive at a conclusion. If in Edinburgh and Berlin light hair and complexions are more common, and dark people predominate in Paris, it may well happen that the disease is more frequent in persons of one complexion in the two former places, and in those of a different complexion at the other, and yet the opinion of Mr. Lloyd may be correct." P. 145.

In his description of the *Comparative Pathology of Scrofula*, in the following chapter, Dr. G. quotes chiefly from the elaborate work of Heusinger, reviewed in a recent number (for April last) of this Journal. He might have also consulted with advantage many of the numbers of that clever periodical, the Veterinarian, which seems to have escaped his notice.

So much for the Pathology of Scrofula, the consideration of which occupies the *first* part of the present volume; the *second* part is devoted to the subject of its Treatment. Unfortunately authors have but little, that is valuable, to tell us upon this head, save in the way of repetition of what their predecessors have already said. We may briefly notice one or two points, which may have the attraction of novelty at least for some of our readers.

As a curious specimen of very fanciful therapeutic speculation, we may point to Ascherson's and Klencke's theories of the action of Cod-liver Oil in scrofulous disease:

"He finds, that when albumen comes in contact with fluid fat, a coagulation takes place in the former, in consequence of which, a sacculated membrane or cell is formed, containing a molecule of the latter. He thus considers that the use of cod-liver oil in scrofula, may consist in its power of enabling, mechanically, the excess of albumen in the blood to be worked up into blood globules. The action of cod-liver oil is, in all probability, as a tonic, from the resinous principle which it contains; by stimulating animal heat; occasionally by acting as an aperient, and also, as a deobstruent, more particularly by increasing the quantity of urine. A more specific power is claimed for it by Klencke, who makes its usefulness to be owing to its supplying the deficiency of the fatty principles of the bile, which, according to him, are not excreted in sufficient quantity in scrofula, but remain in the organ, constituting the fatty liver so often found in this disease."* P. 244.

The *modus operandi* of Iodine is thus attempted to be explained by Dr. Glover:

"When we consider the probable connexion of the secondary digestion of the tissues of which the principles of the urine are the chief results, with the state of the blood and the respiration, we may understand the important part which the use of a remedy like iodine may play in the treatment of such a disease as scrofula: 1st. In quickening the powers of absorption and getting rid of the effused albumen, (and tubercle is composed chiefly of albumen,) where this is not in such a form as to preclude all action of the kind; and 2nd, in removing the excess of albuminous substance in the blood. Again, we deem it by no means an improbable supposition that the chief seats of the formation of urea, may be in the lymphatic glands of the general system. This substance is not formed in the kidneys, as we know by the experiment of Prevost and Dumas. Now, is it not very probable that the lymphatic glands may play such a part on fluids absorbed from the digestion of the tissues, as there is reason to attribute to those of the mesentery and others in the course of the chyle, upon this fluid?"† P. 257.

---

* We read of the following highly philosophic and most instructive (!) experiment by one of these German theorists:—

"Klencke shaved two dogs, and subjected them to frictions of the oil; after three weeks of this treatment the animals were killed, and 'the bile contained as much fat, the chyle as many corpuscles with nuclei, the animal was in general in as good condition, as if he had taken the oil internally.'" P. 274.

† "Dr. Prout attributes the urea of the urine to the transformation of the

Medical chemists come in for a gentle rebuke in the following passage; premising that our author has, with much shew of reasoning, pointed out that there is a marked analogy between Chlorine, Iodine, and Bromine, and their respective salts, in their action upon the system.

" Since the first discovery of iodine in the mineral waters of Piedmont, by M. Cantu, and the almost constant detection of this substance, or bromine, in every mineral water containing a large quantity of chlorides, in which the research has been undertaken; a ludicrous degree of importance has been attached to the existence of minute quantities of bromine and iodine in waters known to be highly charged with the alkaline and earthy muriates; as if such difference existed between the activity of chlorides, and bromides, iodides, as that a proportions of $1\frac{1}{8}$th of bromine, and $\frac{1}{8}$th of iodine, to $189\frac{1}{2}$ grs. of chloride of sodium, alleged to be found by Mr. West in the Woodhall, (or Iodine!) spa, the water containing at the same time the chlorides of lime and magnesium, could be of such great consequence. But the greatest absurdity is the making bromine and iodine figure in the analyses uncombined, at all; as if these powerful electro-negative elements could remain for a *single moment* in such waters in a free state. According to Mr. West's analysis of this very water, there exists in it a quantity of bicarbonate of soda! Now we believe that bromide of sodium might be used as a condiment instead of the chloride." P. 271.

In taking leave of Dr. Glover, we feel much pleasure in expressing our opinion that his work reflects credit alike upon him as the author, and upon the Medical Society of London, in having selected it for the Fothergillian prize. It displays excellent scholarship, and an ardent zeal in the pursuit of professional knowledge.

gelatinous tissues, and the uric acid to the metamorphosis of the albuminous constituents of the body; but this is manifestly erroneous, on account of the small proportion which uric acid bears to urea in the urine, while the albuminous tissues far exceed the gelatinous. It is also opposed to the views of Liebig, who considers urea to be the ultimate stage of the transformation, and itself arising from a change produced upon the uric acid." P. 7.

